# Oral Administration of a Seed-based Bivalent Rotavirus Vaccine Containing VP6 and NSP4 Induces Specific Immune Responses in Mice

**DOI:** 10.3389/fpls.2017.00910

**Published:** 2017-05-31

**Authors:** Hao Feng, Xin Li, Weibin Song, Mei Duan, Hong Chen, Tao Wang, Jiangli Dong

**Affiliations:** ^1^State Key Laboratory of Agrobiotechnology, College of Biological Sciences, China Agricultural UniversityBeijing, China; ^2^State Key Laboratory of Agrobiotechnology and National Maize Improvement Center of China, Department of Plant Genetics and Breeding, China Agricultural UniversityBeijing, China

**Keywords:** rotavirus, plant-based vaccine, oral vaccine, VP6, NSP4

## Abstract

Rotavirus is the leading cause of severe diarrheal disease among newborns. Plant-based rotavirus vaccines have been developed in recent years and have been proven to be effective in animal models. In the present study, we report a bivalent vaccine candidate expressing rotavirus subunits VP6 and NSP4 fused with the adjuvant subunit B of *E. coli* heat-labile enterotoxin (LTB) in maize seeds. The RT-PCR and Western blot results showed that VP6 and LTB-NSP4 antigens were expressed and accumulated in maize seeds. The expression levels were as high as 0.35 and 0.20% of the total soluble protein for VP6 and LTB-NSP4, respectively. Oral administration of transgenic maize seeds successfully stimulated systemic and mucosal responses, with high titers of serum IgG and mucosal IgA antibodies, even after long-term storage. This study is the first to use maize seeds as efficient generators for the development of a bivalent vaccine against rotavirus.

## Introduction

As transgenic technology advanced over the past decades, plant-based expression systems have experienced rapid development. Viral antigens, antibodies, cytokines and other functional proteins and growth factors have been expressed in tobacco, potatoes, tomatoes, maize and rice (da Cunha et al., 2014; Streatfield et al., 2015). Viral subunits produced by plants are promising vaccine candidates due to the following advantages: large amounts of antigen can be produced at relatively low cost and are easily scaled up to field levels; they are safe and are not contaminated with human viruses; post-translational modification is efficient; and oral administration of edible plant-based vaccines can induce mucosal immune responses, which is the primary barrier against viral infection, and systemic immune responses (Goldstein and Thomas, 2004). The first plant-based vaccine was developed in 1992 by Mason and colleagues, who expressed the hepatitis B surface antigen (HBsAg) in tobacco leaves (Mason et al., 1992). Since then, numerous viral antigens have been produced in plants, such as the cholera toxin B-subunit (CTB), C4V3 epitopic protein of human HIV, M2e peptide of H5N1 and VP1 of FMDV (Zhang et al., 2010; Chan and Daniell, 2015). However, exogenous proteins usually accumulate at relatively low levels when target genes are integrated into the nuclear genome. Plastid transformation exhibits significantly higher expression levels; however, like the bacterial expression system, antigens produced in chloroplasts do not generally undergo post-translational modifications, such as glycosylation (Chan and Daniell, 2015). Thus, plant seeds, especially from rice and maize, offer another option for protein level improvement. Seeds have protein bodies (PBs) and protein storage vacuoles (PSVs) where exogenous proteins can accumulate and avoid hydrolysis (Takaiwa et al., 2015). The MucoRice-based expression system has high levels of target protein, high heat stability, long term storage stability and an efficient immune response (Nochi et al., 2007; Tokuhara et al., 2013). These studies demonstrated the advantages of seed-based expression systems. As one of the most important foods and feed, maize seeds have also been used to generate subunit vaccines. Many proteins have been successfully expressed in maize, such as CTB, subunit B of *E. coli* heat-labile enterotoxin (LTB), HBsAg, NP of H3N2, G protein of rabies virus and S protein of TGEV (Lamphear et al., 2002; Hayden et al., 2012; Karaman et al., 2012; Loza-Rubio et al., 2012; Nahampun et al., 2015).

There are currently two licensed vaccines against rotavirus worldwide: the monovalent vaccine Rotarix and the pentavalent vaccine RotaTeq. Both vaccines are live oral vaccines aimed at conferring comparable protection against natural rotavirus infection. The implementation of vaccines against severe rotavirus has resulted in a protective efficacy of 72–100% in developed countries. However, neither of the two vaccines performed as expected in developing countries where they are most needed. There, the efficacy of the vaccines in preventing rotavirus diseases was approximately only 49–72% (Yen et al., 2014). Rotashield, the first licensed rotavirus vaccine, is associated with intussusception, which is also a concern with the two current vaccines. Unfortunately, it was recently shown that Rotarix and RotaTeq may also cause intussusception in infants after vaccination in different countries and regions; however, the benefits still far outweigh the risks (Escolano et al., 2015; Yung et al., 2015; Stowe et al., 2016). More evidence is required to demonstrate the association of intussusception with the currently available vaccines. The contamination of live vaccines by other viruses is also a concern. In fact, some reports have indicated that two vaccines carrying porcine circovirus may infect humans (Baylis et al., 2011; McClenahan et al., 2011; Gilliland et al., 2012). More research for the development of new rotavirus vaccine is required before the licensed vaccines were proved to be safe and efficient among developed and developing countries. Because previous live virus vaccines have been shown to have many disadvantages, vaccines employing rotavirus subunits and virus-like particles have emerged as appealing candidates.

The rotavirus particle is a three layered particle composed of six structural proteins: the inner capsid layer consists of VP1 and VP3, surrounded by VP2 dimmers; the intermediate layer is formed by VP6 trimmers; and the outer layer consists of VP4 and VP7, which determine the P and G serotypes (Jayaram et al., 2004). The VP6 protein constitutes 51% of the total virion mass. It is highly conserved and contains the group and subgroup determinants (Tang et al., 1997). VP6 expressed in either bacterial or baculovirus expression systems can self-assemble into trimers, DPLs and tubular structures depending on the pH, ionic strength and divalent cation concentration (Mellado et al., 2009; Bugli et al., 2014; Pastor et al., 2014). Antibodies against VP6 can interfere with the viral replication cycle at the beginning of the intercellular phase through the inhibition of viral transcription (Feng et al., 2002). A recent study also indicated that VP6 may act as an adjuvant (Blazevic et al., 2016). There are six non-structural proteins (NSP1 through NSP6) involved in virus infection and replication (Hu et al., 2012). Rotavirus non-structural protein 4 (NSP4) was the first enterotoxin identified and plays a role in rotavirus replication, transcription and morphogenesis. NSP4 has the ability to mobilize Ca^2+^ and induces age- and dose-dependent diarrhea (Dong et al., 1997). It can also interact with DLPs to participate in the viral cycle (Hu et al., 2012). The adjuvant activity of NSP4 was also recently reported (Kavanagh et al., 2010). VP4 and VP7 are targets of neutralizing antibodies. VP6 and NSP4 stimulate the immune response during rotavirus infection and induce non-neutralizing antibodies (Jalilvand et al., 2015).

VP4, VP7, and VP6 are three important structural proteins that have been expressed in tobacco, potatoes, alfalfa and *C. amaranticolor* (Wu et al., 2003; Dong et al., 2005; Zhou et al., 2010; Lentz et al., 2011). In addition, NSP4 has also been expressed in transgenic potatoes (Yu and Langridge, 2001). It has been shown that all these plants are efficient producers of rotavirus subunits. The transgenically expressed antigens retained immunogenicity and could induce immune responses when orally administered to mice. With production exceeding 700 million metric tons per year, maize has become the world’s most important food and feed crop (Ranum et al., 2014). Rotavirus group A may have zoonotic potential to transmit between humans and animals (Cook et al., 2004). The development of a maize-based rotavirus vaccine is of great value in preventing rotavirus infection in both humans and animals.

In the present study, two rotavirus subunits, VP6 and NSP4, were successfully expressed in maize seeds together with the fused adjuvant LTB for the first time. Oral administration of maize seeds or purified protein induced both systemic and mucosal responses in mice, demonstrating the efficiency of this seed-based candidate.

## Materials and Methods

### Codon Optimization and Synthesis of the Target Gene

The 1194-bp coding sequence of VP6 from the human rotavirus group A strain (GenBank accession no. AF260931) was codon optimized for efficient expression in maize (GenBank accession no. KY075639). Numerous codons in the VP6 coding sequence were modified to yield a sequence that is consistent with the codon usage of Zea may^1^. Moreover, sequence analysis revealed that there are six potential polyadenylation signal sequences, including AATAAA, AATGAA, AATAAT, AATATT, AATTAA, AATAAG, AACCAA and GATAAA, and four mRNA destabilizing ATTTA motifs along the original VP6 coding sequence. These sequences may potentially destabilize mRNA and reduce gene expression. Therefore, all these sequences were modified to allow efficient transcription and translation. The GC content of the modified gene was increased from 33 to 57%, which coincides with the GC content in highly expressed maize genes (55%). The amino acid sequence encoded by the modified gene (*sVP6*) was identical to that of the original *VP6*. In addition, the synthesized gene contained a plant translation initiation sequence (Kozak sequence, ACCATGG) at the 5′ end of the coding region and a SEKDEL endoplasmic retention signal at the C terminus.

A second sequence, *sLTB-NSP4*, was also synthesized (GenBank accession no. KY075638). This gene is a fusion of *E. coli LTB* without the signal peptide coding sequence (Kang et al., 2003) and the 66-bp DNA fragment of rotavirus non-structural protein 4 (*NSP4*, encoding 114–135 amino acid). The synthesized genes also contained the Kozak sequence and the ER retention signal.

All sequences were synthesized and cloned into the pMD18-T vector by Generay (Shanghai Generay Biotech Co., China).

### Vector Construction and Maize Transformation

The synthesized *sVP6* or *sLTB-NSP4* was then subcloned into the pMD18-T vector under the control of maize 27-kD γ-zein promoters and terminators via enzyme digestion and ligation, respectively. The individual expression cassette was then digested by *EcoR*I and *Bam*HI or *Xba*I and *Hind*III and inserted into the mini-twin T-DNA binary vector pSB130. For particle bombardment, the linear fragment of the *sVP6* and *sLTB-NSP4* expression cassette was cut off by *Eco*RI and *Pme*I. This fragment, together with the Bar gene from pCambia3301, were combined in a 3:1 molar ratio and coated onto gold microcarriers and then delivered into Hi-II immature embryos via particle bombardment according to the methods reported by Klein et al. (1988). Each shot contained about 1 μg DNA fragments (target gene fragment and marker gene fragment in total). The regeneration of maize plants from embryonic callus was performed according to the methods described by Songstad et al. (1996).

### PCR Analysis of Transgenic Plants

Genomic DNA was isolated from maize leaves using the cetyltrimethylammonium bromide (CTAB) method described previously (Allen et al., 2006). Approximately 250 ng of genomic DNA were used as the template in the PCR mixture. Gene-specific primers for *sVP6*, *sLTB-NSP4* and *Bar* were used to detect the target genes (Supplemental Table 2). The PCR cycling condition was 94°C for 30 s, 55°C for 30 s and 72°C for 30 s or 50 s for 30 cycles. The PCR products were analyzed by electrophoresis on 1.0 or 1.5% agarose gels. The plasmids used for transformation served as a positive control, and genomic DNA from untransformed maize leaves was used as a negative control.

### RNA Extraction and Gene Expression Analysis

For the analysis of target gene transcription, seeds from transgenic maize or the negative control at 15 dap were used for RNA extraction. Total RNA was isolated using TRIzol reagent according to the manufacturer’s instructions (Invitrogen, Carlsbad, CA, United States). Then, 2 μg of total RNA was reverse-transcribed into cDNA with M-MLV reverse transcriptase (Promega, Madison, WI, United States). RT-PCR was performed using SYBR Premix Ex Taq (TaKaRa) on a CFX-96 Real-Time System (Bio-Rad). Gene specific primers were used to test the expression of target genes. The PCR program was as follows: 94°C for 30 s followed by 34 cycles of 94°C for 10 s, 56°C or 58°C for 10 s and 72°C for 30 s or 50 s. The PCR product was examined on a 1.0 or 1.5% agarose gel.

### Purification of VP6 and LTB-NSP4 from Bacteria and Maize Seeds

For the expression of VP6 and LTB-NSP4 in bacteria, complete CDS of VP6 were cloned into pGEX4t-1 vector and the LTB-NSP4 were cloned into pET30a vector, and transformed into E. coli BL21 (DE3). As GST-VP6 and HIS-LTB-NSP4 mainly exist in the inclusion body, the purification of the two proteins were performed according to methods reported by Schoner et al. (1985) and Hunkapiller and Lujan (1986) with modification. In brief, bacteria were collected and resuspend by lysis buffer (50 mM Tris-HCL, 1 mM EDTA, 100 mM NacCl, pH 8.0) plus 8 mg lysozyme and 4 mg deoxycholic acid. The suspension was stirring at 4°C for 20 min, adding Triton X-100 to a final concentration of 2% and then stirring at 37°C for another 20 min. After sonication, NaCl was added to 0.5 M and samples were centrifuged. The precipitates were resuspended by lysis buffer containing 2% Triton X-100. Samples were centrifuged and washed by distilled water twice. Then the precipitates were resuspended by 4 M urea and used for SDS-PAGE. The gel was stained by 0.25 M KCl and the corresponding bands were cut off and determined by Western blot. The gel containing GST-VP6 and HIS-LTB-NSP4 were collected by electrophoresis in Tris-Glycine buffer in a dialysis bag. After remove the gel, the proteins were dialyzed in PBS buffer containing urea (4, 2, 1, 0.5, 0.25, and 0.25 M) and finally without urea. The GST-VP6 and HIS-LTB-NSP4 proteins were concentrated using ultrafiltration tubes and stored at -80°C. To extract total soluble protein from maize seeds, about 100 mg grounded mazie seed powder was defatted twice using hexyl hydride. Extraction buffer [(12.5 mM sodium borate (pH 8.5), 100 mM sodium chloride, 0.1% SDS, 0.1% Triton X-100] was added and the samples were incubated at 37°C on a shaker incubator for 6 h. Then samples were centrifuged at 10,000 *g* for 10 min twice and the supernatant was stored at -80°C. SDS-PAGE result of purified proteins from bacteria and maize seed are shown in Supplemental Figure 1. The endotoxin levels were measured using the toxinsensor^TM^ chromogenic limulus amebocyte lysate (LAL) endotoxin assay kit according to the manufacturer’s instructions (GenScript, United States, L00350). The standard curve and endotoxin levels are given in Supplemental Figure 1. A 0.033EU/ml endotoxin standard was used as contol.

### Quantification of VP6 and LTB-NSP4 Protein in Maize Seeds

The quantity of total soluble proteins was monitored by BCA Protein Assay Kit (Tiangen Biotech, PA115). Antigen concentration in transgenic maize seed was quantified by an ELISA method. In brief, total protein extracts (100 μl/well) were added into a 96-well plate. Serial dilution of purified GST-VP6 and HIS-LTB-NSP4 were used as standards. The plate was incubated overnight at 4°C followed by washing with PBST buffer. Then samples were blocked using 3% BSA at 37°C for 2 h. After washing by PBST, samples were incubated with anti-VP6 or anti-LTB-NSP4 antibody (1:5,000) at 37°C for 1 h. After washing, samples were incubated with the anti-rabbit secondary antibody (1:10,000, 100 μl/well) at 37°C for 1 h. After washing, TMB substrate was added to each well and absorbance was measured using a plate reader at 450 nm. Concentration of VP6 and LTB-NSP4 were determined based on known concentration of standard curves (Supplemental Figure 2). Because standard curves were generated by GST- or HIS- tagged proteins, the original concentration read from the curves means GST-VP6 or HIS-LTB-NSP4 concentrations, and it should be converted to VP6 or LTB-NSP4 according the molecular weight. VP6 is about 42 kDa and GST is about 23 kDa, if the GST-VP6 concentration is *Y*1 and the VP6 concentration is *Y*2, then *Y*2 can be calculated by: *Y*2 = *Y*1 × 42/(42+23). Similarly, the LTB-NSP4 concentration can be calculated by: *Y*4 = *Y*3 × 15/(15+7).

### Western Blot

To examine the expression of target proteins, we carried out a Western blot assay. Maize seed protein was extracted as described above. Total soluble protein was extracted from the maize leaves, roots or stems using protein extraction buffer as previous study reported (Dong et al., 2005). For Western blot, samples from bacteria or plants were boiled for 10 min after adding 4× SDS sample buffer and then loaded onto a 10% or 15% SDS-PAGE gel and run for 1.5–2 h. At the end of the run, one gel was stained with Coomassie blue. Proteins from the other gel were transferred onto a nitrocellulose membrane under constant current. The target proteins were examined using specific antibodies and corresponding secondary antibodies. The results were detected by adding substrate onto the membrane and visualized using the Tanon 5200 imaging system. Because GST- and HIS- tagged proteins were used as positive controls, their molecular weights always higher than antigens purified from maize seeds.

### Tissue-Specific Expression Analysis

To demonstrate the endosperm-specific expression of target genes, we carried out RT-PCR and Western blot analyses. Samples from roots, stems, leaves and seeds at 15 dap were used for RNA and total soluble protein extraction. RT-PCR and Western blot analyses were performed as described above. Gene-specific primers were used to amplify certain genes, including sVP6, LTB-NSP4, Bar and β-actin. Antibodies against VP6, LTB-NSP4 and β-actin were used as the primary antibodies, with a dilution of 1:5000 (for anti-VP6 and anti-LTB-NSP4) and 1:3000 (for anti-β-actin). The expression of β-actin served as an internal control.

### Oral Immunization and ELISA

Sixty-six-week-old female BALB/c mice were fed under controlled conditions and divided into 6 experimental groups. Each group that contained 10 mice was immunized orally at weekly intervals over a 4-week period (days 0, 7, 14, and 21) with non-transgenic maize powder (group 2) or purified seed proteins (group 1), purified VP6 and LTB-NSP4 from *E. coli* (group 3), transgenic maize powder (group 5, 6) or purified transgenic seed proteins (group 4). The mice in group 4 were orally immunized with 7 mg of purified protein from maize seeds containing 25 μg of VP6 and 16 μg of LTB-NSP4, while the mice in group 5 were gavaged with seed powder from newly harvested maize containing approximately 25 μg of VP6 and 14 μg of LTB-NSP4. Group 6 was immunized with seed powder from maize stored under normal conditions for 2 years containing antigens comparable to group 5. Maize seed powder was resuspended with 1.1 ml PBS buffer and a single dose gavage was done by two separated gavage with a 2 h interval. The negative controls, groups 1 and 2, were orally gavaged with equal amounts of purified protein or maize powder from untransformed maize plants, while group 3 was gavaged with 25 μg of VP6 protein and 16 μg of LTB-NSP4 protein purified from *E. col*i.

Saliva was collected 1 and 3 weeks post-initial immunization from each animal for the analysis of anti-VP6 and anit-LTB-NSP4 IgA. To access serum IgG, blood samples were collected via retro-orbital bleeding on day 36. On day 60, the mice in each group were sacrificed, and the small intestines were homogenized in 0.01 M PBS to measure mucosal IgA antibody levels.

The antibody titers were quantified by ELISA according to previously described methods (Dong et al., 2005). The antibody titers were determined as the reciprocal of the highest antibody dilution yielding an OD492 that was greater than or equal to twice the mean OD492 value of antibody from samples from the negative controls. The results are presented as the arithmetic means ± SD.

BALB/c mice were obtained from the Academy of military medical sciences. Animal immunization, management and sample collection were conducted by trained personnel following the guidelines of China Agricultural University Laboratory Animal Welfare and Animal Experimental Ethical Committee. All animal procedures were approved by the China Agricultural University Laboratory Animal Welfare and Animal Experimental Ethical Committee.

### Antibodies

The commercial antibodies used in this study were as follows: primary antibody anti-β-actin (CWBIO, CW0264M) was used to detect the internal control β-actin; anti-mouse IgG (KPL, 074-1806) and goat anti-mouse IgA (Sigma, A4789) secondary antibodies were used to detect serum IgG and mucosal IgA. The anti-VP6 polyclonal antibody was prepared in a previous study (Dong et al., 2005). The anti-LTB-NSP4 polyclonal antibody was prepared from rabbit serum immunized with purified HIS-LTB-NSP4 protein by Beijing Protein Innovation Co., Ltd.

### Statistical Analyses

Arithmetic mean titers of IgG and IgA from groups immunized with transgenic seed powder and purified antigens from bacteria or transgenic maize were compared to those of the control group using Student’s *t*-test in the SPSS statistical software package. *P* < 0.05 was considered significantly different.

## Results

### Synthesis of a Codon-Optimized sVP6 Gene and a LTB-NSP4 Fusion Gene

To improve the expression of VP6, the coding sequence from human group A rotavirus was optimized according to the codon usage preference of Zea may. The GC content was increased from 33 to 57% to match the genes that were highly expressed in maize. In addition, four possible mRNA degradation signals (ATTTA motif) and six potential polyadenylation signals were modified to ensure transcription efficiency (Supplemental Table 1). The entire amino acid sequence of the codon-optimized *sVP6* was identical to the original *VP6* gene to ensure that the transgenically expressed protein retained the antigenicity of the native VP6 protein.

**FIGURE 1:**
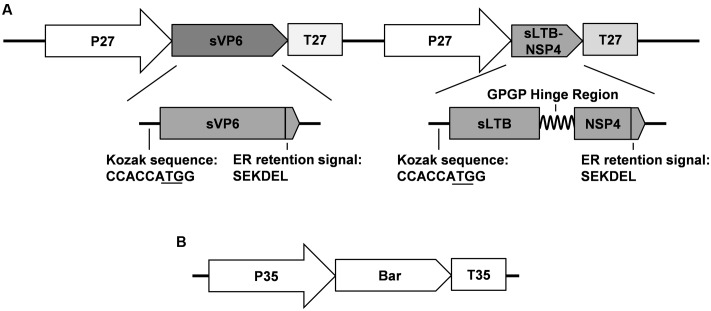
Schematic map of DNA fragments used for maize transformation. **(A)** Structure of target genes frangment. P 27: promoter of maize 27 KD γ-zzein; T 27: terminator of maize 27 KD γ-zein; sVP6: synthesized rotavirus VP6 gene; sLTB-SNP4: synthesized fusion gene encoding the B subunit of *E. coli* heat-labile enterotoxin and the 114–135 peptide of rotavirus NSP4; **(B)** Structure of marker gene fragment. P 35: cauliflower mosaic virus 35S promoter; T 35: 35S: cauliflower mosaic virus 35S terminator; Bar: coding sequence of phosphinothricin acetyltransferase.

To improve the immunogenicity of the plant-based vaccine, we synthesized the *sLTB-NSP4* fusion gene, the encoding subunit B of *E. coli* heat-LTB and the 114–135 peptide of the rotavirus non-structural protein 4 (NSP4). A GPGP hinge region was fused between the LTB and NSP4 peptide to improve the flexibility of the protein (**Figure 1A**). The NSP4 peptide has been reported to be highly immunogenic (Yu and Langridge, 2001). LTB was proven to be an efficient adjuvant for the delivery of antigens (da Hora et al., 2011). The fusion protein is a rotavirus antigen and a mucosal adjuvant.

Each synthesized gene contains the Kozak sequence at its 5′ terminal to improve the translation efficiency and the SEKDEL ER retention signal at the C-terminus to stabilize the protein and confine it to the ER (**Figure 1A**). Proteins restricted to the ER can avoid being glycosylated by the Golgi apparatus with plant-specific α-1,3-fucose and β-1,2-xylose residues that might be immunogenic in humans (Garcia-Casado et al., 1996; Yang et al., 2008). Furthermore, ER and its derived protein body (PB-I) can prevent the hydrolysis of exogenous proteins and improve their accumulation (Takaiwa et al., 2015).

### Vector Construction and Maize Transformation

To construct the vector for maize transformation, *sVP6* and *sLTB-NSP4* were inserted into the same T-DNA under the control of the maize 27-kD γ-zein promoter and terminator, respectively (**Figure 1A**). The 27-kD γ-zein was reported to be one of the most abundant zeins and is expressed only in the endosperm (Ueda and Messing, 1991). The target gene can be restricted to the endosperm by the control of this promoter. This makes the maize-based plant bioreactor safe and efficient.

**FIGURE 2:**
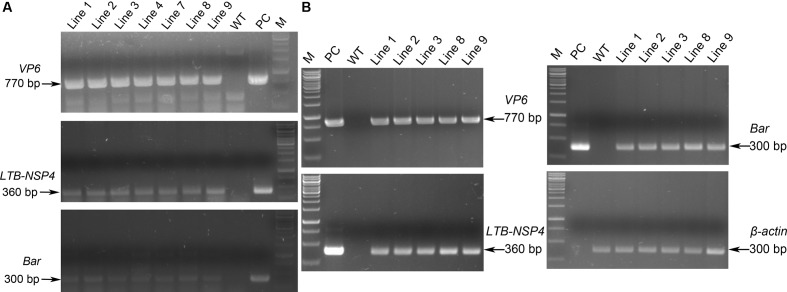
PCR and RT-PCR analysis of transgenic maize. **(A)** PCR identification of transgenic maize. Genomic DNA was used as template. WT: wild type maize plant; Line 1, 2, 3, 4, 7, 8, 9: transgenic maize lines; PC: positive control. Arrows on the left indicate the positive bands. **(B)** RT-PCR analysis of the expression of exogenous genes. Total RNA was extracted from developing seeds. WT: wild type maize plant; Line 1, 2, 3, 8, 9: transgenic maize lines; PC: positive control. β-*actin* was used as internal control. Arrows on the right indicate the positive bands.

To generate transgenic maize plants, we cut off the expression cassettes of *sVP6* and *LTB-NSP4*, and this linear fragment together with the *Bar* gene from pCambia3301 (**Figure 1B**) were transformed into the embryogenic callus by particle bombardment. Genomic DNA from leaves of regenerated seedlings was used as a template tested by PCR. We obtained 9 positive transgenic lines that showed specific bands indicative of *sVP6*, *sLTB-NSP4* and *Bar* (**Figure 2A**). The transcription of exogenous genes was tested by RT-PCR using total RNA extracted from developing seeds (15 dap). The results showed that target genes were expressed in the seeds of the transformed maize plants but not wild type plants (**Figure 2B**). These results demonstrate that the exogenous genes were integrated into the nuclear genome of the transgenic maize plants and successfully expressed.

### *VP6* and *LTB-NSP4* Are Efficiently Expressed in Transgenic Maize

**FIGURE 3:**
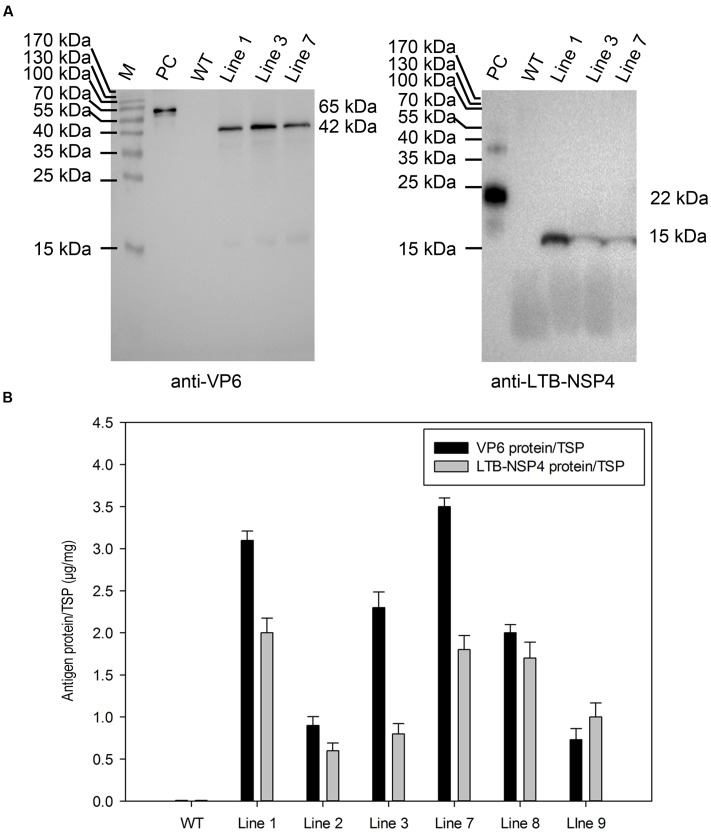
Analysis of protein accumulation in transgenic maize seeds. **(A)** Western blot analysis of VP6 and LTB-NSP4 protein in the seeds of transgenic maize. Line 1, 3, 7: transgenic maize lines; WT: wild type maize plant; PC: recombinant GST-VP6 and HIS-LTB-NSP4 protein expressed in *E. coli* were used as positive controls. The expected molecular weights of target proteins are indicated on the right and antibodies used in the detection are showed below. **(B)** Quantitation of VP6 and LTB-NSP4 proteins expressed in transgenic maize seeds by ELISA. WT: wild type maize plant; Line 1, 2, 3, 7, 8, 9: seed samples from individual transgenic maize plants. TSP: total soluble protein.

Western blot analysis further confirmed expression at the protein level. Total soluble protein was extracted from plant seeds and examined using antibodies against VP6 and LTB-NSP4. The result showed that a 42-kDa band representing VP6 and a 15-kDa band representing the LTB-NSP4 fusion protein were detected in transgenic maize but not in the negative control (**Figure 3A**). This indicated that the target antigens efficiently accumulated in the maize seeds.

Next, we carried out an ELISA assay to quantify the accumulation of target proteins. The result showed that the accumulation of codon-optimized VP6 in seeds ranged from 0.07 to 0.35% TSP, with the highest expression of approximately 9 μg per seed. The expression level of the non-optimized LTB-NSP4 fusion protein ranged from 0.05 to 0.20% TSP and the highest expression was approximately 5 μg per seed (**Figure 3B**). All antigens were expressed at relatively high levels, demonstrating that the maize seed is an efficient expression system. The endosperm-specific 27-kD γ-zein promoter and subcellular localization may also contribute.

### Target Gene Expression Is Localized in Maize Seeds

**FIGURE 4:**
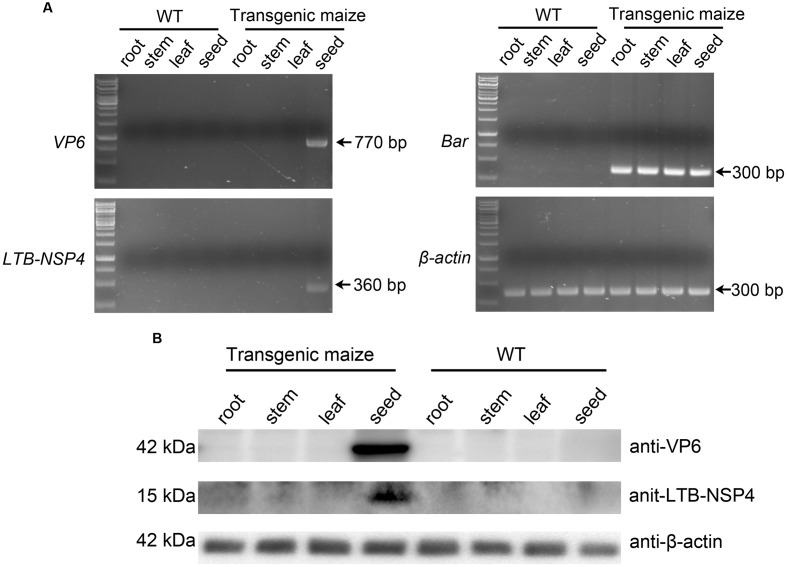
Tissue specific expression of target genes. **(A)** RT-PCR examination of the specificity of target genes. Total RNAs were extracted from root, stem, leaf and developing seed of wild type (WT) or transgenic maize plants. Maize β-*actin* was used as internal control. Arrows on the right indicate the positive bands. **(B)** Western blot analysis of target protein accumulation specificity. Total soluble proteins were extracted from root, stem, leaf and seed of wild type (WT) or transgenic maize plants. Antibodies used are indicated on the right and expected molecular weight of target proteins are indicated on the left. β-actin was used as loading control.

The target genes were under the control of the 27-kD γ-zein promoter, which exhibits tissue-specific expression activity in the endosperm. Therefore, the VP6 and LTB-NSP4 proteins were expected to be restricted to the seeds. We performed RT-PCR to confirm this assumption. Total RNA extracted from roots, stems, leaves and the developing seeds were reverse-transcribed into cDNA and used as templates. The *Bar* gene under the control of the CaMV 35s promoter and the β-*actin* housekeeping gene were expressed in all transgenic maize samples tested. *SVP6* and *sLTB-NSP4* were detected only in the transgenic developing seeds. None of the genes were expressed in the roots, stems or leaves (**Figure 4A**). Western blot analysis further confirmed this result. We tested proteins extracted from roots, stems, leaves and seeds of transgenic and wild type maize using antibodies against VP6 and LTB-NSP4. VP6 and LTB-NSP4 proteins accumulated in the seeds and were hardly detected in the other organs, while the internal control β-actin was ubiquitously expressed (**Figure 4B**). Taken together, these results demonstrate that *VP6* and *LTB-NSP4* genes are expressed efficiently and are localized in the transgenic maize seeds.

### Oral Administration of Bivalent Rotavirus Vaccine Induces Immune Responses in Mice

**FIGURE 5:**
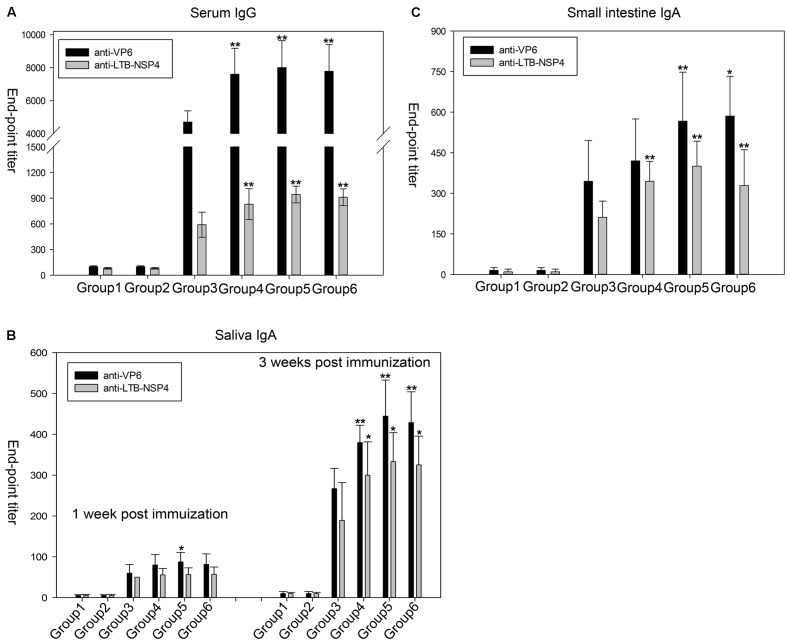
Anti-VP6 and anti-LTB-NSP4 antibody titers determined by ELISA in mice after oral immunization. **(A)** VP6-specific and LTB-NSP4-secific serum IgG examined at 36 day post the initial immunization. **(B)** Specific IgA titers against VP6 and LTB-NSP4 in the small intestine at 60 day post the initial immunization. **(C)** Saliva IgA titers against VP6 and LTB-NSP4 determined at 1 and 3 week post the initial immunization. All groups immunized with VP6 and LTB-NSP4 antigens induced significantly higher antibody titers compared with the two groups gavaged with wild type maize seeds (*P* < 0.01), and the significances are not indicated in the figures. The significances showed in the figures above represent certain group compared to group 3. ^∗^*P* < 0.05 and ^∗∗^*P* < 0.01. Group 1: mice gavaged with purified protein from wild type maize seed; Group 2: mice gavaged with wild type maize seed powder; Group 3: mice gavaged with purified protein from *E. coli*; Group 4: mice gavaged with purified protein from transgenic maize seed; Group 5: mice gavaged with seed powder from newly harvested transgenic maize; Group 6: mice gavaged with seed powder from transgenic maize stored for 2 years. The results are presented as the arithmetic means ± SD.

To test the immunogenicity of the seed-based rotavirus subunits, BALB/c mice were gavaged with purified proteins from transgenic maize seeds (containing 25 μg of VP6 and 16 μg of LTB-NSP4, group 4), transgenic maize seed powder (group 5) or seed powder from transgenic maize stored for 2 years (group 6). We used purified proteins from bacteria containing equal amounts of antigens as a positive control (group 3). Purified proteins (group 1) or seed powder (group 2) from wild type maize served as a negative control. Systemic IgG and mucosal IgA titers were quantified by ELISA. Mice that received either purified protein or maize powder from wild type maize seeds did not exhibit any antibody responses, while the other groups gavaged with VP6 and LTB-NSP4 either from transgenic maize or bacteria showed significantly higher antibody titers (*P* < 0.01, **Figure 5**).

In group 4 the serum IgG titers ranged from 1/5000 to 1/10000 and 1/500 to 1/1000 against VP6 and LTB-NSP4, respectively. The VP6-specific serum IgG titers in mice gavaged with maize powder (group 5) or maize powder stored for 2 years (group 6) ranged from 1/6000 to 1/10000, and the IgG titers against LTB-NSP4 in these groups ranged from 1/800 to 1/1000. All three of these groups exhibited higher serum IgG titers against VP6 and LTB-NSP4 than the group that received purified proteins from bacteria orally (group 3). The anti-VP6 titer ranged from 1/4000 to 1/6000, and the anti-LTB-NSP4 titer ranged from 1/500 to 1/800 (**Figure 5A**). The VP6-specific IgG titer was comparable to findings from our previous study that reported serum IgG titers ranging from 1/6000 to 1/9600 after mice were immunized with transgenic alfalfa expressing rotavirus VP6 (Dong et al., 2005). The LTB-NSP4-specific IgG titer was higher than in mice that received transgenic potato containing CTB-NSP4 orally (serum IgG of approximately 1/125) (Yu and Langridge, 2001). This finding suggested that the seed-based bivalent vaccine efficiently induced a systemic response, even after long-term storage.

Saliva IgA was induced 1 week after the first immunization and increased after sequential immunization. Three weeks after the first immunization, groups gavaged with purified proteins from either seeds or seed powder showed significantly stronger immune responses against VP6 and LTB-NSP4 than group 3 (**Figure 5B**). The IgA titers of group 5 and group 6 ranged from 1/400 to 1/600 for VP6 and 1/200 to 1/300 for LTB-NSP4, respectively. The IgA titer against VP6 was comparable to those determined by Zhou et al. (2010), who reported antibody titers against VP6 ranging from 1/360 to 1/600 after mice were gavaged with *C. amaranticolor* expressing VP6.

Oral administration of VP6 and LTB-NSP4 also stimulated small intestine mucosal IgA. We measured small intestinal IgA 60 days after the first immunization. VP6-specific IgA in group 4 was comparable to that of group 3. Groups gavaged with maize powder (group 5 and group 6) developed significantly higher VP6-specific IgA titers than group 3. Antibody titers against LTB-NSP4 in group 4, group 5, and group 6 were also higher than in group 3 (**Figure 5C**). The small intestinal IgA titer against VP6 was higher than that reported in our previous study (Dong et al., 2005), in which the IgA titer ranged from 1/320 to 1/640, and was comparable to that reported by Zhou et al. (2010), with IgA titer of 1/300 to 1/720. The small intestinal IgA titer against LTB-NSP4 was higher than that reported by Yu and Langridge (2001), with antibody titer about 1/15. This finding suggested that the bivalent vaccine efficiently induced mucosal responses when administered orally.

Our results demonstrate that oral administration of the seed-based bivalent vaccine efficiently stimulates both systemic and mucosal responses. Additionally, the immunogenicity of the vaccine was not affected by long-term storage. The seed-based vaccine is a promising vaccine candidate for immunization against rotavirus.

## Discussion

Over the past few decades, the use of plants in the production of antigens has emerged as a promising expression system that has many advantages, including safety and cost-effectiveness. It has been shown that orally administered plant-based vaccines effectively stimulate both systemic and mucosal immune responses. We previously reported a plant-based monovalent subunit vaccine candidate expressing the middle layer rotavirus protein VP6 in transgenic alfalfa (Dong et al., 2005). Although the expression level was relatively high (0.28% of TSP), the net production of the antigen was quite low in plant leaves. In the current study, we successfully generated transgenic maize that expressed high levels of target proteins in seeds through codon optimization, tissue specific expression and other means. The amount of codon-optimized sVP6 reached 0.35% of TSP or 9 μg per seed. The accumulation of non-optimized sLTB-NSP4 reached 0.20% of TSP or 5 μg per seed (**Figure 3B**). The biomass yield of maize is larger than all other grains, including rice, wheat and barley. Compared to maize leaves and roots, the seed is more stable and has higher production yields, making it an efficient target protein generator (Loza-Rubio et al., 2012; Nahampun et al., 2015). It has also been suggested that antigens produced in the seeds are safer (Nahampun et al., 2015). The production of rabies G proteins in maize seeds was approximately 25 μg/g of seed weight (Loza-Rubio et al., 2012). The accumulation of H3N2 NP in maize seeds ranged from 8 to 35 μg/g (Nahampun et al., 2015). The highest accumulation for VP6 and LTB-NSP4 were 28 and 17 μg/g in the current study. The expression levels measured in our study were comparable to these researches and may have been even higher if we consider that multiple antigens were expressed simultaneously in one seed. To the best of our knowledge, this is the only study to report the expression of the rotavirus subunit in maize seeds. Our success in expressing VP6 and LTB-NSP4 in maize seeds extends the research on rotavirus subunit vaccines in plants.

As the middle layer protein, VP6 and the non-structural protein NSP4 stimulate non-neutralizing antibodies that could not clear virus particles through immune clearance. The protective mechanisms of the VP6 and NSP4 vaccine against rotavirus infection are currently incompletely understood. Both have been shown to induce systemic and mucosal immunity as well as T cell-mediated immune responses involving the production of cytokines. Pastor et al. (2014) reported that high protection against rotavirus infection was correlated with high anti-vp6 IgG titers when mice were immunized subcutaneously with different assemblies of VP6. More studies have indicated that protection against virus challenge is the result of stimulation of secretory IgA (sIgA) by mucosal administration of VP6, rather than IgG. Anti-VP6 IgA does not exhibit any neutralizing activity, but polymeric IgA can move the rotavirus into the gut lumen via transcytosis from the basolateral membrane to the apical membrane of enterocytes (Burns et al., 1996; Corthesy et al., 2006). This result also demonstrated the important role of mucosal immunity in rotavirus defense. However, other studies have demonstrated that VP6-induced T cell responses and cytokine secretion rather than antibody induction were critical for rotavirus defense in B cell-deficient mice (Choi et al., 1999; Franco and Greenberg, 1999). Studies investigating NSP4 reported similar results demonstrating the involvement of antibody responses, T cells and cytokines (Enouf et al., 2001; Ray et al., 2003; Vizzi et al., 2005; Choi et al., 2006). In this study, oral administration of a maize seed-based vaccine expressing VP6 and LTB-NSP4 fusion protein stimulated both systemic and mucosal immunity as indicated by the induction of serum IgG and mucosal IgA (**Figure 5**). Mice immunized with antigens from maize seeds showed significantly higher IgG and IgA titers than those immunized with purified protein from *E. coli*. This might be due to the protection of antigens in the gastrointestinal tract by secondary metabolites of plant like polyphenolics, or, more likely, by the well-known function of plant cells called bioencapsulation (Kong et al., 2001; Chan and Daniell, 2015). Furthermore, VP6 and LTB-NSP4 were located in the endoplasmic reticulum in this study. Proteins with ER retention signals expressed in maize or rice seed usually accumulate in the ER and its derived PB-I (Takaiwa et al., 2015). This is important in the prevention of post-translational N-glycosylation by the Golgi apparatus, which contains the carbohydrate groups α-1,3-fucose and β-1,2-xylose. These types of N-glycosylations in plants are potential human immunogens and may cause allergic reactions (Garcia-Casado et al., 1996). PB-I also provided further protection against gastrointestinal digestion because proteins deposited in PB-I are more resistant to digestive enzymes. To our surprise, antibody titers against VP6 were always higher than those against LTB-NSP4. This may have resulted from the high immunogenicity of VP6 compared to LTB and the 22 amino acid peptide of NSP4. In fact, VP6 is the most immunogenic subunit of rotavirus, and several antigenic epitopes have been identified (Kohli et al., 1993).

We previously showed that oral administration of transgenic alfalfa containing VP6 stimulated a serum IgG titer of 1/8000 and a small intestinal IgA titer of 1/500, and passive immunity was observed in the offspring (Dong et al., 2005). Zhou et al. (2010) reported that mice immunized orally with VP6 expressed in *C. amaranticolor* leaves induced IgG and IgA antibodies and further protected suckling mice against rotavirus challenge; the antibody titers were 1/6000 for serum IgG and 1/400 for intestinal IgA. Yu and Langridge (2001) demonstrated that oral administration of CTB-NSP4 transgenic potato tuber induced a serum titer of approximately 1/125 and an intestinal IgA titer of approximately 1/15. Moreover, newborns were also protected from rotavirus challenge. Compared to these reports, the antibody titers reported in the current study were relatively high (**Figure 5**), particularly sIgA, which is the leading exemplar of mucosal immunity and has been proven to prevent rotavirus replication and clear virus particles during transcytosis. This may have been due to a stronger immune response elicited by high concentrations of antigens in maize seeds. Secondary metabolites and PB-I may also contribute to the presentation of antigens. Previous studies have shown that VP6 and NSP4 can induce passive immunity and protect suckling mice against rotavirus challenge. In the current study, we propose that a maize seed-based vaccine may also confer passive protection because the antibody titers here were comparable to, or even higher than, those reported previously.

Our previous work showed that oral administration of pBsVP6-transgenic alfalfa induces specific antibody responses and protects mice against rotavirus challenge. However, the antigen delivered by oral immunization in that study had to be combined with the mucosal adjuvant CpG ODN 1826. This additional step markedly reduced the efficacy of the vaccine and increased the cost. Here, we report that the newly developed vaccine solved this problem by expressing the non-toxic adjuvant LTB fused to the NSP4 antigen (**Figure 1**). The efficacy of subunit vaccines is usually associated with co-administration of the proper adjuvant. The heat-LT of *E. coli* was proven to be an effective adjuvant but is unsuitable for human use due to its toxicity. Therefore, several non-toxic derivatives of LT have been developed, including the subunit B (LTB), and non-toxic LT mutants, such as LTR72, LTK63 and LTR192G (da Hora et al., 2011). The B subunit of LT can form a pentameric structure with a central cavity. This structure has been directly related to the binding activity of the GM1 receptor. The interaction of LTB pentamers and GM1 can enhance the presentation of LTB-conjugated antigens and stimulate B and T cell-related immune responses. Yu and Langridge (2001) reported that CTB fused to NSP4 induced a stronger immune response than the co-administration of CTB and NSP4. The result was the same when LTB was used as the adjuvant. Zhou et al. (2009) showed that titers of antibodies against multi-epitopes of *Helicobacter pylori*, especially mucosal IgA, in mice immunized with the HUepi-LTB fusion protein were higher than in those immunized with HUepi alone or HUepi in combination with LTB. Moreover, Martin and Nashar (2013) demonstrated that the fusion of LTB-gag p24 resulted in better T cell stimulation than LTB and the gag p24 mixture during the immune response against HIV. In the present study, the induction of both specific serum IgG and mucosal IgA demonstrated the efficiency of LTB as an adjuvant (**Figure 5**). The increased VP6 antibody titers relative to LTB-NSP4 fusion protein may have been due to differences in the immunogenicity of the antigens as discussed above. Our results also suggested that transgenic maize retained high immunogenicity even after 2 years of storage at ambient conditions (**Figure 5**). The expression of antigens together with the adjuvant makes the maize seed-based vaccine a ready-to-use candidate against rotavirus.

This study presents a bivalent subunit vaccine together with the non-toxic adjuvant LTB as a candidate against rotavirus. This candidate possesses all the advantages of plant-based vaccines and can induce both mucosal and systemic immune responses. VP6 and NSP4 are two promising antigens in subunit rotavirus vaccine research; however, they have never been used in combination. Therefore, our study provides a foundation for the development of a VP6-NSP4 bivalent vaccine against rotavirus. However, some areas still require improvement. First, VP6 or NSP4 expressed in transgenic alfalfa, C. *amaranticolor* or potato has been shown to stimulate passive immunity and protect suckling mice against rotavirus challenge (Yu and Langridge, 2001; Dong et al., 2005; Zhou et al., 2010). Further research is required to investigate whether the maize seed-based rotavirus vaccine can induce passive immunity in animal models. Second, we used two individual DNA segments for maize transformation (**Figure 1**) so that the target and marker genes could undergo segregation during meiosis (Prakash et al., 2009; Wang et al., 2013). In subsequent experiments, we will focus on screening for marker-free transgenic maize lines. The development of a marker-free vaccine candidate will improve its safety and be more easily accepted.

## Author Contributions

JD and TW conceived and designed research. HF, XL, WS, MD, and HC conducted experiments. HF analyzed data and wrote the manuscript. All authors read and approved the manuscript.

## Conflict of Interest Statement

The authors declare that the research was conducted in the absence of any commercial or financial relationships that could be construed as a potential conflict of interest.

## Footnotes

1 http://www.kazusa.or.jp/codon/
