# Supplementary material for: Oral Administration of a Seed-based Bivalent Rotavirus Vaccine Containing VP6 and NSP4 Induces Specific Immune Responses in Mice
Source: Front Plant Sci. 2017 May 31;8:910. doi: 10.3389/fpls.2017.00910 (PMC5449476; doi:10.3389/fpls.2017.00910)
Supplement: Supplementary file 1 [file Table_1.DOCX]

**Table S1 Comparison of the *VP6* gene and modified *sVP6* gene**

*VP6* 1 ATGGAGGTTCTGTACTCATTGTCAAAAACTCTTAAAGATGCTAGAGATAAAATTGTTGAA 60

|||||||| || ||| | || || ||||| ||||| ||||| || || || |||||

*sVP6* 1 ATGGAGGTGCTCTACAGCCTCTCCAAGACTCTCAAAGACGCTAGGGACAAGATCGTTGAG 60

*VP6* 61 GGTACATTATATTCCAATGTTAGCGATCTCATTCAACAATTTAATCAAATGATAATGACT 120

|| || | ||| ||| || ||||| || || || || || || || ||||| |||||

*sVP6* 61 GGCACCCTGTATAGCAACGTGAGCGACCTGATCCAGCAGTTCAACCAGATGATTATGACC 120

*VP6*  121 ATGAATGGAAATGACTTTCAAACTGGAGGAATTGGTAATTTACCAGTTAGAAATTGGATT 180

||||| || || ||||| || || || || || || ||| | || || || || |||||

*sVP6* 121 ATGAACGGCAACGACTTCCAGACCGGCGGCATCGGCAATCTGCCGGTGAGGAACTGGATC 180

*VP6*  181 TTTGATTTTGGTCTATTAGGTACAACACTTTTAAATTTGGATGCTAATTATGTTGAAAAT 240

||||| || || || | || || || || | || |||| || || || || || |||

*sVP6* 181 TTTGACTTCGGCCTGCTCGGCACTACTCTGCTGAACCTGGACGCGAACTACGTGGAGAAT 240

*VP6*  241 GCAAGAACTACGATTGAATATTTCATTGATTTTATTGATAATGTATGTATGGATGAAATG 300

|| || || || || || || ||||| || || || || || || || ||||| || |||

*sVP6* 241 GCCAGGACCACCATCGAGTACTTCATCGACTTCATCGACAACGTGTGCATGGACGAGATG 300

*VP6*  301 GCAAGAGAGTCTCAAAGAAATGGAGTAGCTCCACAATCTGAAGCGTTGAGGAAATTATCA 360

|| | ||||| || || || || || || || ||| || || |||||||| | ||

*sVP6* 301 GCGCGCGAGTCCCAGAGGAACGGCGTTGCCCCCCAAAGCGAGGCCCTGAGGAAACTGTCC 360

*VP6*  361 GGCATTAAATTTAAGAGGATAAATTTTGATAATTCATCAG--AATATATAGAAAATTGGA 418

||||| || || |||||||| || || || || || ||| | || || || || ||||

*sVP6* 361 GGCATCAAGTTCAAGAGGATTAACTTCGACAA--CAGCAGCGAGTACATCGAGAACTGGA 418

*VP6*  419 ATCTACAAAATAGAAGACAGCGTACTGGATTTGTCTTTCATAAACCTAATATATTTCCAT 478

| || || || || | || | || || || || || ||||| || || || ||||| |

*sVP6* 419 ACCTGCAGAACAGGCGCCAAAGGACCGGCTTCGTGTTCCATAAGCCGAACATCTTTCCGT 478

*VP6*  479 ACTCAGCTTCGTTCACTTTGAATAGATCTCAACCAATGCATGATAATTTGATGGGAACTA 538

|| || || ||||| | || | || || |||||||||||||| | ||||| ||||

*sVP6* 479 ACAGCGCGTCCTTCACGCTCAACCGCTCCCAGCCAATGCATGATAACCTCATGGGCACTA 538

*VP6*  539 TGTGGCTTAATGCTGGATCGGAGATACAAGTAGCTGGTTTTGATTATTCATGTGCTATAA 598

||||||| ||||| || || ||||| ||||| ||||| || || || || || || |

*sVP6* 539 TGTGGCTGAATGCGGGCTCCGAGATCCAAGTGGCTGGCTTCGACTACAGCTGCGCCATTA 598

*VP6*  599 ACGCACCAGCAAATATACAGCAGTTTGAACATATTGTACAGCTTAGACGTGCACTAACCA 658

|||| || || || || || ||||| || || || || || || | | || || ||||

*sVP6* 599 ACGCGCCTGCCAACATCCAACAGTTCGAGCACATCGTGCAACTCCGCAGGGCGCTCACCA 658

*VP6*  659 CAGCTACTATAACTTTGTTACCTGATGCAGAAAGATTTAGTTTTCCAAGAGTTATCAATT 718

| || || || || || | || || || || | ||| || || | |||||||||

*sVP6* 659 CCGCCACGATCACACTGCTCCCGGACGCTGAGCGCTTTTCCTTCCCTCGCGTTATCAATA 718

*VP6*  719 CGGCTGACGGCGCAACTACATGGTTTTTTAATCCAGTCATTCTAAGACCAAATAATGTAG 778

|| |||||||| || |||||||| || || || ||||| || || || || || || |

*sVP6* 719 GCGCGGACGGCGCTACCACATGGTTCTTCAACCCGGTCATCCTGAGGCCCAACAACGTCG 778

*VP6*  779 AAGTAGAATTTTTGTTGAATGGACAAATTATTAACACATATCAGGCTAGATTTGGTACTA 838

| || || || || | || || || || || ||||| || ||||| || ||||| || |

*sVP6* 779 AGGTGGAGTTCCTGCTCAACGGCCAGATCATCAACACCTACCAGGCGAGGTTTGGCACGA 838

*VP6*  839 TTATCGCAGGAAATTTTGATACAATTCGATTGTCATTTCAGTTAATGCGTCCACCAAATA 898

||||||| || ||||| || || || | |||| || ||| | ||| | ||||| ||||

*sVP6* 839 TTATCGCCGGCAATTTCGACACCATCAGGCTGTCCTTCCAGCTGATGAGGCCACCCAATA 898

*VP6*  899 TGACACCAGCTGTTAACGCATTATTTCCGCAAGCGCAACCTTTTCAACACCATGCAACAG 958

|||| ||||| || ||||| | || ||||||||||| || || || ||||| || || |

*sVP6* 899 TGACGCCAGCCGTGAACGCCCTGTTCCCGCAAGCGCAGCCATTCCAGCACCACGCTACCG 958

*VP6*  959 TTGGACTCACATTACGCATTGAATCTGCTGTCTGTGAATCAGTGCTTGCGGATGCGAATG 1018

| || ||||| | ||||| ||| || || |||||| || || || || || || |

*sVP6* 959 TCGGCCTCACCCTGCGCATCGAAAGCGCGGTGTGTGAAAGCGTTCTCGCCGACGCCAACG 1018

*VP6*  1019 AAACTCTGTTAGCGAATGTGACCGCAGTGCGTCAAGAATATGCTATACCAGTTGGACCGG 1078

| || ||| | || ||||||||||| ||||| || |||||||| || || ||||| ||||

*sVP6* 1019 AGACGCTGCTCGCCAATGTGACCGCCGTGCGCCAGGAATATGCCATCCCGGTTGGCCCGG 1078

*VP6*  1079 TTTTTCCACCAGGCATGAATTGGACTGAGCTAATTACTAACTATTCACCATCGAGAGAAG 1138

| ||||| ||||||||||||||||| ||||| || ||||| || || || | ||||

*sVP6* 1079 TGTTTCCCCCAGGCATGAATTGGACCGAGCTGATCACTAATTACAGCCCGTCCCGCGAAG 1138

*VP6*  1139 ATAACCTGCAACGTGTCTTTACAGTAGCTTCCATTAGAAGCATGTTGATTAAGTGA 1194

|||| |||||| | || || || || |||||||| | |||| |||| ||||||

*sVP6* 1139 ATAATCTGCAAAGGGTTTTCACCGTGGCTTCCATCCGCTCCATGCTGATCAAGTGA 1194
