# Supplementary material for: Oral Administration of a Seed-based Bivalent Rotavirus Vaccine Containing VP6 and NSP4 Induces Specific Immune Responses in Mice
Source: Front Plant Sci. 2017 May 31;8:910. doi: 10.3389/fpls.2017.00910 (PMC5449476; doi:10.3389/fpls.2017.00910)
Supplement: Supplementary file 2 [file Table_2.DOCX]

**Table S2** **Sequence of oligonucelotides used in experiments**

| Primer name | Oligonucleotide sequence |
| --- | --- |
| 27 kD Pro-F | 5’-GAATTCCGTCCCGCGTCAATATTATTAAAAAAC-3’ |
| 27 kD Pro-R | 5’-CCATGGTGGTGTCGATCGGGTTCTTCTGCGCT-3’ |
| VP6-F-Nco I | 5’-CCATGGAGGTGCTCTACAGCCTCTC-3’ |
| VP6-R-Sac I | 5’-GAGCTCTCACTTGATCAGCATGGAGCGGAT-3’ |
| LTB-F-Nco I | 5’-CCATGGCTCCTCAGTCTATTACAGA-3’ |
| LTB-R-Sac I | 5’-GAGCTCTCACAGCTCGTCCTTCTCGCTTAT-3’ |
| VP6-F | 5’-TCCAAGACTCTCAAAGACGCTAG -3’ |
| VP6-R | 5’-AGGAACTCCACCTCGACGTTGTT-3’ |
| LTB-NSP4-F | 5’-CAGTCTATTACAGAACTATGTTC-3’ |
| LTB-NSP4-R | 5’-CAACCTGTTCAATTTCACGAGTTG-3’ |
| *Zm*actin-F | 5’-ATTGAGCATGGCATTGTCAACAAC-3’ |
| *Zm*actin-R | 5’-CTTCATAGATTGGAACCGTGTGGCTC-3’ |
| Bar-F | 5’-GTCTGCACCATCGTCAACCACTA-3’ |
| Bar-R | 5’-TCTTGAAGCCCTGTGCCTCCA-3’ |
