# Supplementary figures and images for: Oral Administration of a Seed-based Bivalent Rotavirus Vaccine Containing VP6 and NSP4 Induces Specific Immune Responses in Mice
Source: Front Plant Sci. 2017 May 31;8:910. doi: 10.3389/fpls.2017.00910 (PMC5449476; doi:10.3389/fpls.2017.00910)

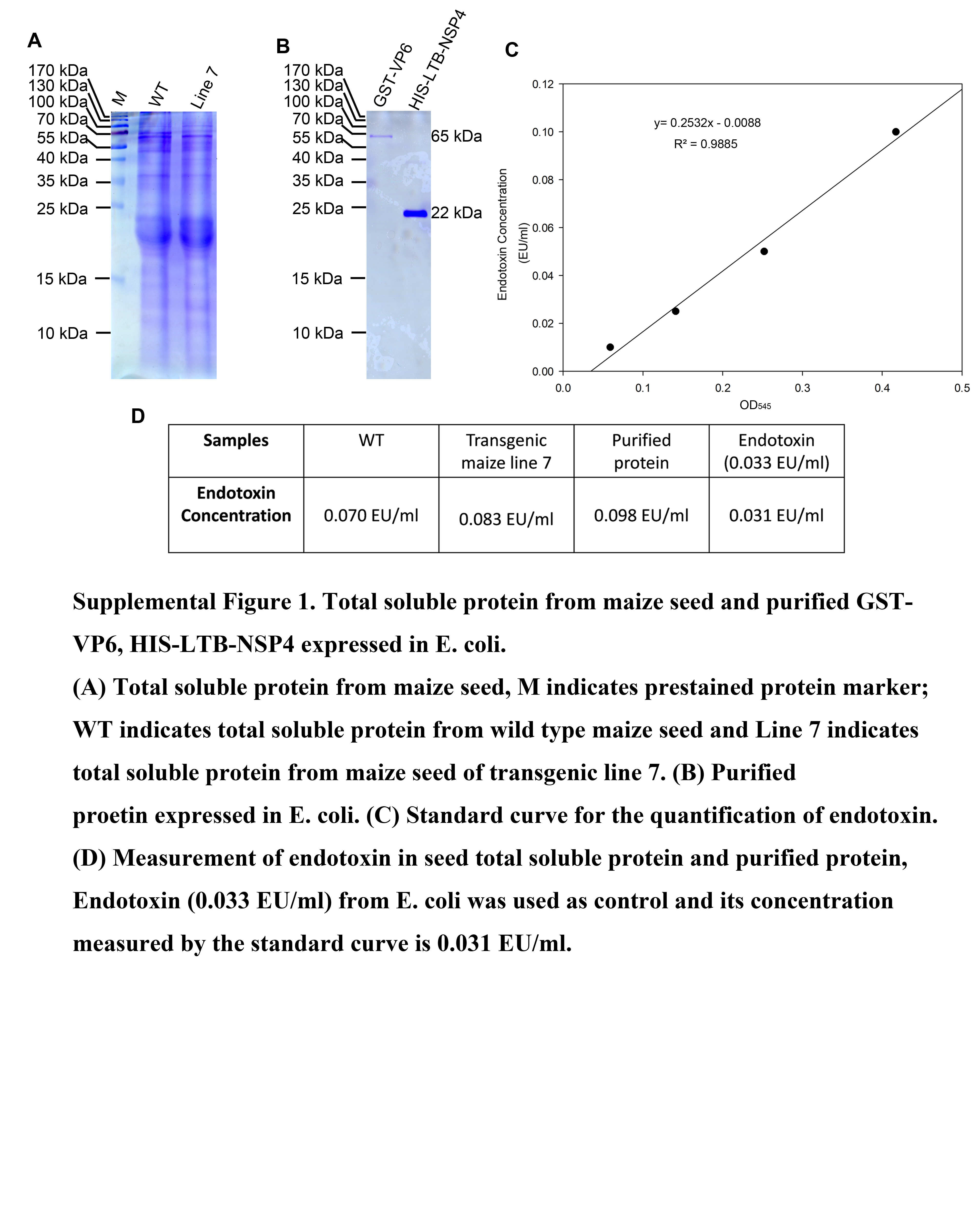

Supplement: Supplementary file 3 [file Image_1.TIF]

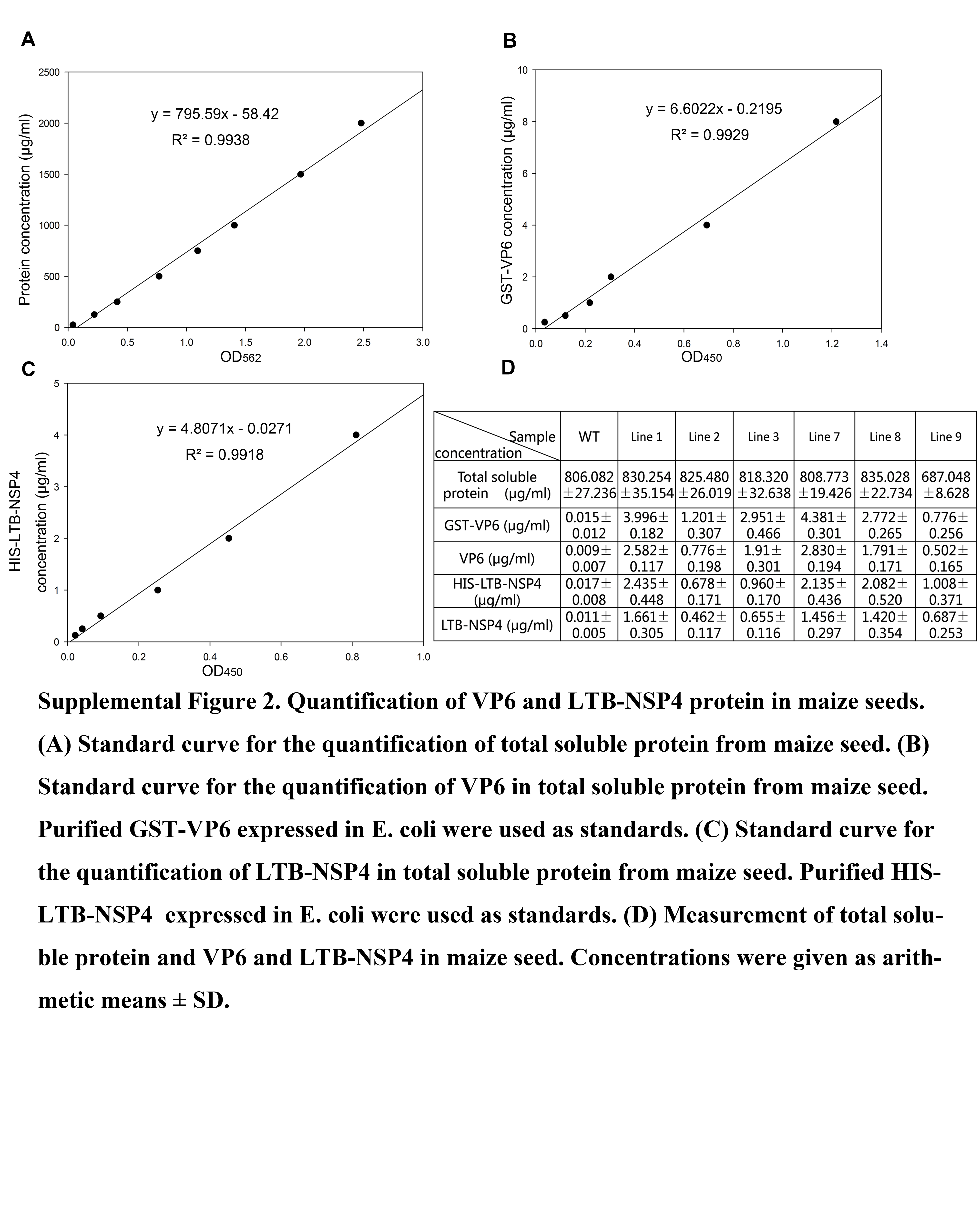

Supplement: Supplementary file 4 [file Image_2.TIF]
